# The web-based ASSO-food frequency questionnaire for adolescents: relative and absolute reproducibility assessment

**DOI:** 10.1186/1475-2891-13-119

**Published:** 2014-12-17

**Authors:** Anna Rita Filippi, Emanuele Amodio, Giuseppe Napoli, João Breda, Antonino Bianco, Monèm Jemni, Laura Censi, Caterina Mammina, Garden Tabacchi

**Affiliations:** Department of Sciences for Health Promotion and Mother Child Care “G. D’Alessandro”, University of Palermo, Via Del Vespro 133, 90127 Palermo, Italy; Division of Non-communicable Diseases and Life-Course, World Health Organization Regional Office for Europe, UN City, Marmorvej 51, DK - 2100 Copenhagen, Denmark; Sport and Exercise Sciences Unit, University of Palermo, Via E. Duse, 2, 90146 Palermo, Italy; School of Science, University of Greenwich, Centre for Sport and Human Performance, Central Avenue, Chatham Maritime, Kent, ME4 4TB UK; Agricultural Research Council, Food and Nutrition Research Centre (CRA-NUT), Via Ardeatina, 546, 00178 Rome, Italy

**Keywords:** Food frequency questionnaire, Reproducibility, Adolescent, Nutrient, Intake

## Abstract

**Background:**

A new food frequency questionnaire (FFQ) has been recently developed within the Italian Adolescents and Surveillance System for the Obesity prevention (ASSO) Project; it was found to be appropriate for ranking adolescents in food and nutrient levels of intake. The aim of this study was to assess the relative and absolute reproducibility of the ASSO-FFQ for 24 food groups, energy and 52 nutrients.

**Methods:**

A test-retest study was performed on two ASSO-FFQs administered one month apart of each other to 185 adolescents, aged 14–17 and attending secondary schools in Palermo (Italy). Wilcoxon test assessed differences in median daily intakes between the two FFQs. Agreement was evaluated by quintiles comparison and weighted kappa. Intraclass Correlation Coefficients (ICC) and Bland-Altman method assessed the relative and absolute reliability respectively.

**Results:**

Significant difference (p < 0.05) in median intakes was found only for bread substitutes, savoury food, water, soft drinks, carbohydrates and sugar. The subjects classified into the same or adjacent quintiles for food groups ranged from 62% (white bread) to 91% (soft drinks); for energy and nutrients from 64% (polyunsaturated fatty acids) to 90% (ethanol). Mean values of weighted kappa were 0.47 and 0.48, respectively for food groups and nutrients. Fair to good ICC values (>0.40) were assessed for thirteen food groups, energy and forty-three nutrients. Limits of Agreement were narrow for almost all food groups and all nutrients.

**Conclusions:**

The ASSO-FFQ is a reliable instrument for estimating food groups, energy and nutrients intake in adolescents.

**Electronic supplementary material:**

The online version of this article (doi:10.1186/1475-2891-13-119) contains supplementary material, which is available to authorized users.

## Background

Public health studies need reliable and valid measures of daily food and nutrients intake in adolescents. Among the several methods to assess dietary intake, food frequency questionnaires (FFQs) are commonly used because of their low cost and ease of use [1, 2]. However, the FFQ’s reproducibility is of prime concern [3]. The concept of reproducibility refers to the consistency of data obtained in more than one administration of the same instrument to the same subject at different times [4]. Two types of reliability have been identified, i.e. the relative reliability and the absolute one [5]. Relative reliability is about the consistency of the individual’s position within a group with regards to the others [6]. Basically, food, energy and nutrients intake can vary widely with time, so precision at individual level could be poor even if there is a good agreement of the mean intakes. Therefore, also the absolute reliability, i.e. the degree to which repeated measurements vary for individuals [7], should be taken into account.

Different FFQs have been validated and have been shown to be reliable [4, 8–12], but the need of a web-based, more user-friendly, fast and cost-effective tool has been recently highlighted [13]. To this purpose, the ASSO-FFQ has been developed within the Adolescents and Surveillance System for the Obesity prevention (ASSO) Project, financially supported by the Italian Ministry of Health. It is a web-based questionnaire included in the ASSO-NutFit (Nutrition & Fitness) software that allows obtaining a database on food groups, energy and nutrients intake in adolescents. It has been previously validated against a 7-day weighted food record (WFR) (2014, unpublished observations). The validation study revealed that, even though the ASSO-FFQ was not suitable for measuring the absolute intakes of all food groups and nutrients, it was appropriate for ranking adolescents in food and nutrient levels; moreover, type of school, gender, alcohol consumption and between meals were significant explanatory variables of the intake differences between FFQ and WFR, thus influencing the questionnaire validity.

The aim of this study was to assess the relative and absolute reproducibility of the ASSO-FFQ for 24 food groups, energy and 52 nutrients.

## Methods

### Study design and participants

This reproducibility study applied a test-retest design. It was approved by the ethical committee of the Azienda Ospedaliera Universitaria Policlinico "Paolo Giaccone" in Palermo (approval code n.9/2011). All participants were provided with information sheets and had to supply the informed consent signed by their parents before the beginning of the study.

A multistage sampling was used for the selection of subjects participating in the Project: at the first stage, a systematic sampling of 7 out of the 55 public and private high schools of Palermo, stratified per type of school (lyceum, technical and professional institute), was performed; in the second stage, a cluster sampling of classes for each selected school allowed obtaining the sample of students. A subgroup of students was selected for the reproducibility study, on the basis of the type of school and age.

### ASSO-FFQ’s administration

Participants were asked to web-compile two ASSO-FFQs at one month apart of each other, during classroom time and under the supervision of trained teachers, in March and April 2013. The ASSO-FFQ is a self-administered and semi-quantitative questionnaire, asking the portion size and the frequency of consumption over the previous six months. Portion size is assessed through the use of three pictures showing three sizes of the food/beverage (small, medium, large) and of household units; the following frequencies were used to assess the frequency of consumption: never, 1–2 times per month, once per week, 2–4 times per week, 5–6 times per week, once per day, twice per day, 3–5 times per day.

The ASSO-FFQ comprises a total of 106 food items, and requires on average 20 min to be compiled.

Data collected from both FFQs were processed within the ASSO-NutFit software and were transformed into daily energy and nutrients intake by means of the Italian tables of nutrient composition ( http://sito.entecra.it/portale/cra_dati_istituto.php?id=1004&) of the Istituto Nazionale di Ricerca per gli Alimenti e la Nutrizione (INRAN) and of the food composition databases ( http://fnic.nal.usda.gov/food-composition) of the United States Department of Agriculture (USDA), that were included into the software.

In order to facilitate the conversion into nutrients, the 106 food items were combined according to their nutrient composition (see Additional file 1) into 24 food/beverage items that were finally investigated: vegetables, fresh fruit, dried fruit, nuts, legumes, breakfast cereals, white bread, bread substitutes, pasta/rice/couscous, potatoes, sweets, cheeses/yogurt, fishery products, meat, eggs, animal fats, oils, savoury food, water, soft drinks, fruit juice, milk, tea/coffee, alcoholic drinks. Energy and a total of 52 nutrient values were also considered as outcomes: total fat, saturated fatty acids (SFA), myristic acid, palmitic acid, stearic acid, monounsaturated fatty acids (MUFA), oleic acid, polyunsaturated fatty acids (PUFA), linoleic acid, linolenic acid, arachidonic acid, eicosapentaenoic acid (EPA), docohexaenoic acid (DHA), trans fatty acids (TFA), cholesterol, proteins, arginine, cystine, phenylalanine, isoleucine, histidine, leucine, lysine, methionine, tyrosine, threonine, tryptophan, valine, carbohydrates, sugar, fructose, lactose, sucrose, starch, fiber, water, calcium, phosphorus, iron, magnesium, vitamin A RAE (Retinol Activity Equivalents), thiamin, riboflavin, niacin, vitamin B_6_, folate, vitamin B_12_, vitamin C, vitamin D, vitamin E, ethanol, caffeine.

Further indications on the development, data treatment and validation of the ASSO-FFQ are showed in the validation study (2014, unpublished observations).

Web-based data obtained through the ASSO-FFQ’s compilation were automatically included into a database by the ASSO-NutFit software, after performing an automatic checking of data entry.

### Statistical analysis

The obtained database was entered the software STATA/MP 12.1 (StataCorpLP, college Station, TX, USA) and statistical analyses were then performed.

Since the data were not normally distributed, as assessed through the Shapiro-Wilk test, medians and interquartile ranges of food groups, energy and nutrient intakes were carried out on data from the two compiled FFQs. Using the Wilcoxon signed rank test, intake estimates of food groups, energy and nutrients obtained from the FFQs were compared. The proportion of subjects categorized in the same quintile by both the FFQs, in the same or adjacent quintile and in all other quintiles was determined. Weighted kappa was used to express agreement in the classification of individuals and was weighted to take into account the degree of disagreement between the two FFQs. They were compared with the following thresholds [14]: ≤0 = less than chance agreement; 0.01–0.20 = slight agreement; 0.21–0.40 = fair agreement; 0.41–0.60 = moderate agreement; 0.61–0.80 = substantial agreement; 0.81–0.99 = almost perfect agreement.

Intraclass Correlation Coefficient (ICC), one of the most commonly used relative reliability index, was estimated. ICC values were interpreted as follows: ≤ 0.40 = poor reliability; 0.41–0.75 = fair to good reliability; >0.75 = excellent reliability [15].

To describe absolute reliability, Bland-Altman levels of agreement (LOA) were performed according to the following formulation:


where  is the mean difference between the FFQs, *sd* is the standard deviation of the difference between them, *t*_*n* - 1,0.05_ is the value of *t* corresponding to two-sided p-value = 0.05 for *n* – 1 degrees of freedom and  is an adjustment for small sample size.

The 95% LOA proposed by Bland and Altman were showed to check whether the variability and the precision of the ASSO-FFQ’s measurements were related to the size of the intake estimates [16]. LOA by food groups were obtained overlaying the plot of difference versus mean between the two FFQs. The exponentiated mean difference and LOA provided the ratio of intake estimated by the two FFQs: LOA ranging between 50 and 200% indicated an acceptable agreement [17]. ICC and Bland & Altman analyses were performed on log-transformed, energy-adjusted data to achieve normality, taking into account the confounding effect related to the total consumption of energy. Student t test was used to assess mean differences; significant dependence of the difference in intake estimates from the average level of intake was assessed through linear regression.

## Results

Food groups and nutrients intake of 185 male and female adolescents (75% M, 25% F), aged 14–17 (mean 15.9, SD 1.01), was investigated (as shown in Table 1).

**Table 1:** **Sample composition per age and sex**

Age (years)	Females	%	Males	%	Total	%
**14**	9	20%	11	8%	20	11%
**15**	12	26%	26	19%	38	21%
**16**	15	33%	39	28%	54	29%
**17**	10	22%	63	45%	73	39%
**Total**	46	100%	139	100%	185	100%

### Food groups

Food groups’ median intakes, estimated by both the FFQs, are shown in Table 2. Differences between medians were significant (positive) only for bread substitutes, savoury food, water and soft drinks.

**Table 2:** **Median, interquartile range, Wilcoxon test, quintiles comparison, weighted kappa of 24 food groups daily intakes**

	ASSO-FFQ1			ASSO-FFQ2			Difference between medians ^a^	% correct classified	% correct or adjacent classified	% all the others	Weighted kappa
Food groups	Median	First quartile	Third quartile	Median	First quartile	Third quartile					
Vegetables (g)	112.85	48.57	232.86	95.35	29.65	238.57	17.50	33	74	26	0.51
Fresh fruit (g)	150.00	67.15	307.14	150.00	64.29	302.86	0.00	38	77	23	0.58
Dried fruit (g)	0.00	0.00	0.36	0.00	0.00	1.07	0.00	58	83	17	0.21
Nuts (g)	0.21	0.00	0.57	0.00	0.00	0.21	0.21	54	86	14	0.39
Legumes (g)	21.43	10.36	51.43	22.85	8.57	47.15	-1.42	31	64	36	0.33
Breakfast cereals (g)	1.07	0.00	19.29	1.61	0.00	19.29	-0.54	30	82	18	0.47
White bread (g)	47.14	18.57	84.29	45.72	17.14	98.58	1.42	31	62	38	0.33
Bread substitutes (g)	20.64	10.64	44.13	17.25	7.07	37.36	3.39*	29	70	30	0.49
Pasta/rice/couscous (g)	119.29	60.00	216.79	108.57	47.14	192.86	10.72	37	74	26	0.47
Potatoes (g)	69.65	34.29	120.01	60.00	26.43	109.29	9.65	38	69	31	0.48
Sweets (g)	95.40	41.58	188.99	89.16	41.72	172.99	6.24	37	77	23	0.56
Cheeses/yogurt (g)	50.36	15.89	119.29	46.96	16.25	112.14	3.40	40	73	27	0.46
Fishery products (g)	32.22	11.79	75.28	36.43	13.94	72.15	-4.21	41	77	23	0.56
Meat (g)	171.45	104.64	256.08	164.29	96.43	280.72	7.16	36	75	25	0.47
Eggs (g)	8.57	2.14	25.71	8.57	2.14	8.57	0.00	50	70	30	0.41
Animal fats (g)	0.71	0.18	2.14	0.89	0.18	2.32	-0.18	39	73	27	0.56
Oils (g)	36.97	22.51	63.71	34.12	20.37	57.62	2.85	34	70	30	0.42
Savoury food (g)	222.85	122.84	367.85	194.99	107.84	295.69	27.86*	37	72	28	0.41
Water (ml)	4 000.00	2 000.00	6 000.00	3 000.00	1 000.00	4 000.00	1 000.00**	35	63	37	0.32
Soft drinks (ml)	53.57	8.92	254.46	29.65	4.46	153.22	23.92***	39	91	9	0.59
Fruit juice (ml)	85.71	14.28	200.00	35.71	7.14	171.42	50.00	34	68	32	0.51
Milk (ml)	196.43	35.71	250.00	116.07	17.86	250.00	80.36	43	79	21	0.57
Tea/coffee (ml)	35.71	3.58	100.00	39.29	1.79	100.00	-3.58	51	84	16	0.57
Alcoholic drinks (ml)	36.43	7.50	127.85	47.14	11.79	153.57	-10.71	57	89	11	0.66

*P < 0.05; **P < 0.01; ***P < 0.001.

^a^Medians significantly different (Wilcoxon signed rank test for difference) between paired observations.

The percentage of adolescents classified into the same quintiles was 40% on average, ranging from 29% (bread substitutes) to 58% (dried fruit), while the percentage of correctly or adjacent classified ranged from 62% (white bread) to 91% (soft drinks), with a mean value of 75%. The weighted kappa values showed substantial agreement (0.61-0.80) for alcoholic drinks, and moderate agreement between 0.41-0.60 for vegetables, fresh fruit, breakfast cereals, bread substitutes, pasta/rice/couscous, potatoes, sweets, cheeses/yogurt, fishery products, meat, eggs, animal fats, oils, savoury food, soft drinks, fruit juice, milk, tea/coffee. Dried fruit, nuts, legumes, white bread and water showed fair values of kappa (between 0.21 and 0.40); no food groups showed low agreement. The mean kappa value was 0.47.

Poor relative reliability was assessed for dried fruit, nuts, legumes, breakfast cereals, white bread, bread substitutes, pasta/rice/couscous, potatoes, fishery products, eggs, oils, with ICC ≤ 0.40, while for all the other food groups, namely vegetables, fresh fruit, sweets, cheeses/yogurt, meat, animal fats, savoury food, water, soft drinks, fruit juice, milk, tea/coffee, alcoholic drinks, fair to good reliability was observed (ICC > 0.40) (Table 3).

**Table 3:** **Intraclass correlation coefficients, exponentiated mean difference and 95% LOA of food groups daily intake, performed on transformed, energy-adjusted data**

Food groups	ICC	Mean difference (%)	P-value t test	Lower limit (%) ^a^	Upper limit (%) ^a^
Vegetables (g)	0.46	99.97	0.952	89.68	111.44
Fresh fruit (g)	0.54	100.25	0.654	87.74	114.55
Dried fruit (g)	0.03	100.00	0.857	99.51	100.50
Nuts (g)	0.22	100.00	0.797	99.92	100.08
Legumes (g)	0.14	99.97	0.803	97.36	102.66
Breakfast cereals (g)	0.27	99.97	0.639	98.35	101.62
White bread (g)	0.34	99.94	0.765	95.55	104.54
Bread substitutes (g)	0.21	100.06	0.544	97.62	102.57
Pasta/rice/couscous (g)	0.36	100.10	0.606	95.57	104.85
Potatoes (g)	0.37	99.98	0.889	95.83	104.29
Sweets (g)	0.43	99.89	0.639	94.35	105.75
Cheeses/yogurt (g)	0.41	99.89	0.614	95.07	104.97
Fishery products (g)	0.40	99.84	0.152	97.19	102.56
Meat (g)	0.41	99.56	0.040	94.61	104.76
Eggs (g)	0.37	100.02	0.557	99.22	100.83
Animal fats (g)	0.44	100.00	0.827	99.90	100.10
Oils (g)	0.23	99.94	0.398	98.34	101.57
Savoury food (g)	0.41	100.23	0.475	92.81	108.24
Water (ml)	0.47	102.05	0.611	39.63	262.83
Soft drinks (ml)	0.49	100.95	0.241	83.48	122.06
Fruit juice (ml)	0.41	100.59	0.274	88.64	114.15
Milk (ml)	0.56	100.50	0.318	89.30	113.11
Tea/coffee (ml)	0.56	100.09	0.649	95.38	105.04
Alcoholic drinks (ml)	0.51	99.73	0.286	93.99	105.83

^a^Lower and upper Limits Of Agreement estimated through the Bland-Altman method.

Thirteen out of the 24 food groups showed intake estimates from FFQ2 generally lower than those ones from the first administration; however, these differences were significant (p-value < 0.05) only for meat (Table 3).

**Figure 1:**
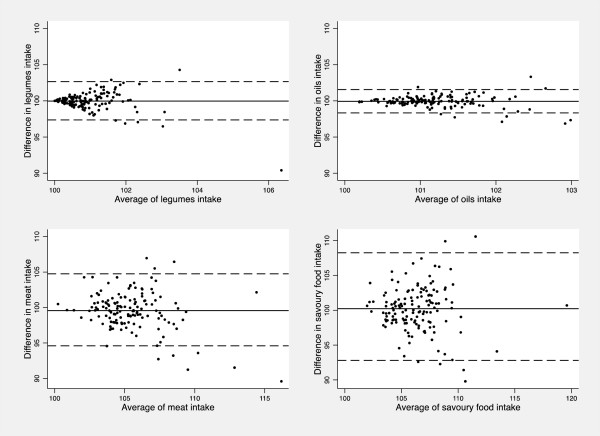
**Bland Altman plots for the reproducibility analysis of legumes, oils, meat and savoury food.** The solid horizontal lines indicate the mean difference (percentage) between the two measures and the broken horizontal lines indicate the lower and upper Limits of Agreement (±t_91;0.025_SDs).

The exponentiated value of mean differences (mean ratio) was 100.15% on average. LOA were within 50% and 200% for food groups, except for water, whose lower and upper limits were 39.63% and 262.83% respectively (Table 3).Only eight out of the 24 food groups showed significant dependence (p-value < 0.05) of the difference in intake estimates from the average level of intake: dried fruit, nuts, legumes, bread substitutes, potatoes, meat, savoury food and fruit juice. As an example, scatter plots with LOA of legumes, oils, meat and savoury food are shown (Figure 1).

### Energy and nutrients

Table 4 shows median intakes of energy and nutrients, estimated by both the FFQs, and the difference between medians. Wilcoxon signed rank test assessed significant differences of median intakes only for carbohydrates and sugar.

**Table 4:** **Median, interquartile range, Wilcoxon test, quintiles comparison and weighted kappa of energy and nutrients daily intakes**

Nutrients	ASSO-FFQ1			ASSO-FFQ2							
	Median	First quartile	Third quartile	Median	First quartile	Third quartile	Difference between medians ^a^	% correctly classified	% correctly or adjacent classified	% all the others	Weighted kappa
Energy (kcal)	3 245.15	2 346.47	4 458.82	2 996.25	1 817.75	4 099.51	248.90	32	69	31	0.38
Total fat (g)	140.39	98.15	200.76	134.06	79.60	185.78	6.33	29	73	27	0.39
SFA (g)	69.23	44.80	171.09	70.41	43.37	165.40	-1.18	41	74	26	0.47
Myristic acid (g)	1.48	1.05	2.30	1.48	0.93	2.13	0.00	31	66	34	0.40
Palmitic acid (g)	18.16	12.63	26.69	17.65	10.23	25.15	0.52	30	72	28	0.45
Stearic acid (g)	8.23	5.92	12.59	7.74	4.77	11.37	0.50	32	73	27	0.46
MUFA (g)	66.32	43.55	95.77	64.69	38.12	91.91	1.63	32	65	35	0.41
Oleic acid (g)	34.80	24.51	50.12	35.18	22.15	48.81	-0.38	29	65	35	0.40
PUFA (g)	26.25	16.07	47.41	25.18	13.94	48.69	1.07	32	64	36	0.32
Linoleic acid (g)	22.83	13.30	42.37	21.47	11.90	41.30	1.36	37	65	35	0.32
Linolenic acid (g)	1.20	0.80	1.66	1.10	0.68	1.54	0.10	33	73	27	0.45
Arachidonic acid (g)	0.16	0.11	0.22	0.14	0.10	0.25	0.02	33	72	28	0.51
EPA (g)	0.08	0.04	0.17	0.09	0.04	0.16	-0.01	39	75	25	0.58
DHA (g)	0.16	0.08	0.34	0.18	0.07	0.33	-0.03	44	75	25	0.62
TFA (g)	0.90	0.58	1.43	0.92	0.54	1.33	-0.02	32	71	29	0.40
Cholesterol (mg)	318.45	227.24	491.17	293.57	184.76	449.79	24.89	32	72	28	0.45
Proteins (g)	118.49	85.74	168.40	112.11	69.29	154.62	6.38	35	70	30	0.45
Arginine (g)	5.18	3.79	7.17	5.22	3.24	7.20	-0.04	32	70	30	0.49
Cystine (g)	1.30	0.97	1.73	1.28	0.77	1.73	0.02	31	69	31	0.49
Phenylalanine (g)	4.24	3.19	5.97	4.19	2.60	5.70	0.05	35	75	25	0.50
Isoleucine (g)	3.98	2.95	5.44	3.67	2.36	5.48	0.31	30	73	27	0.50
Histidine (g)	2.93	2.23	4.13	2.94	1.90	4.13	-0.02	32	71	29	0.49
Leucine (g)	12.88	7.49	23.11	12.79	6.87	24.33	0.10	35	68	32	0.42
Lysine (g)	12.64	6.83	22.55	12.33	6.55	23.53	0.31	32	70	30	0.41
Methionine (g)	2.97	1.97	4.07	2.76	1.87	3.98	0.21	33	73	27	0.51
Tyrosine (g)	3.40	2.57	4.85	3.31	2.13	4.64	0.09	34	72	28	0.51
Threonine (g)	3.58	2.64	5.03	3.58	2.21	4.89	0.00	34	75	25	0.50
Tryptophan (g)	1.45	0.95	1.96	1.35	0.92	1.92	0.10	36	73	27	0.51
Valine (g)	4.68	3.48	6.37	4.26	2.74	6.41	0.43	30	73	27	0.50
Carbohydrates (g)	361.98	260.25	503.68	339.96	202.30	447.99	22.03*	30	69	31	0.36
Sugar (g)	104.97	74.41	152.52	95.27	65.08	133.47	9.71*	28	70	30	0.46
Fructose (g)	13.82	7.74	24.99	12.71	7.14	24.61	1.11	34	75	25	0.52
Lactose (g)	10.17	3.14	13.95	7.22	2.24	13.14	2.95	46	80	20	0.59
Sucrose (g)	11.99	8.44	17.25	11.25	7.22	16.62	0.74	39	77	23	0.54
Starch (g)	101.81	72.13	161.65	100.26	61.52	147.31	1.55	30	67	33	0.41
Fiber (g)	32.00	21.18	42.70	29.03	17.97	40.98	2.97	37	68	32	0.41
Water (ml)	1 342.20	912.43	1 743.27	1 161.84	804.77	1 768.61	180.36	39	75	25	0.48
Calcium (mg)	1 079.31	698.31	1 486.43	979.70	593.06	1 452.65	99.61	31	70	30	0.42
Phosphorus (mg)	1 629.90	1 195.17	2 268.23	1 570.07	995.03	2 134.50	59.83	38	73	27	0.47
Iron (mg)	23.34	15.61	30.55	21.94	13.74	31.80	1.40	35	66	34	0.40
Magnesium (mg)	346.07	239.18	460.32	316.80	205.82	446.47	29.27	40	71	29	0.46
Vitamin A (RAE)	650.08	389.61	1 008.94	613.63	310.69	1 005.83	36.45	31	73	27	0.50
Thiamine (mg)	1.74	1.24	2.51	1.59	1.03	2.45	0.15	39	70	30	0.51
Riboflavin (mg)	2.13	1.51	2.93	2.04	1.34	3.16	0.09	33	70	30	0.46
Niacin (mg)	118.13	78.78	229.63	122.50	67.88	208.29	-4.37	36	78	22	0.57
Vitamin B_6_ (mg)	2.70	1.86	3.81	2.50	1.59	4.02	0.20	37	74	26	0.46
Folate (μg)	264.65	173.93	387.41	236.81	172.02	363.14	27.84	35	70	30	0.46
Vitamin B_12_ (μg)	8.34	5.70	11.38	8.52	4.65	12.51	-0.18	33	78	22	0.56
Vitamin C (mg)	132.87	83.73	190.89	110.75	78.79	179.34	22.13	36	67	33	0.47
Vitamin D (IU)	3.84	2.20	5.92	3.85	1.85	6.28	-0.01	39	75	25	0.60
Vitamin E (mg)	355.96	243.52	452.40	318.30	208.20	476.98	37.66	34	70	30	0.45
Ethanol (g)	1.83	0.37	6.17	1.85	0.37	7.17	-0.01	56	90	10	0.72
Caffeine (mg)	19.81	5.82	40.30	15.57	4.44	36.17	4.25	42	85	15	0.66

*P < 0.05; **P < 0.01; ***P < 0.001.

^a^Medians significantly different (Wilcoxon signed rank test for difference) between paired observations.

SFA: saturated fatty acids; MUFA: monounsaturated fatty acids; PUFA: polyunsaturated fatty acids; EPA: eicosapentaenoic acid; DHA: docohexaenoic acid; TFA: trans fatty acids; RAE: retinol activity equivalents.

The percentage of adolescents classified into the same quintiles was on average 35%, ranging from 28% (sugar) to 56% (ethanol), while the percentage of correctly or adjacent classified ranged from 64% (PUFA) to 90% (ethanol), with a mean value of 72%.

The weighted kappa values showed substantial agreement (0.61-0.80) for DHA, ethanol and caffeine, while ranged between 0.21-0.40 (fair agreement) for energy, total fat, myristic acid, oleic acid, PUFA, linoleic acid, TFA, lysine, carbohydrates, starch, fiber and iron. All the other nutrients showed moderate agreement (between 0.41 and 0.60).

ICC values ranged between 0.21 and 0.40 only for 9 nutrients (total fat, myristic acid, MUFA, oleic acid, PUFA, linoleic acid, cholesterol, starch and iron), while all the other nutrients showed fair to good reliability (ICC > 0.40) (Table 5).

**Table 5:** **Intraclass correlation coefficients, exponentiated mean difference and 95% LOA of nutrients daily intake, performed on transformed, energy-adjusted data**

Nutrients	ICC	Mean difference (%)	P-value t test	Lower limit (%) ^a^	Upper limit (%) ^a^
Total fat (g)	0.36	100.40	0.284	92.31	109.42
SFA (g)	0.50	99.80	0.884	77.11	129.69
Myristic acid (g)	0.33	100.20	0.602	90.48	110.52
Palmitic acid (g)	0.43	100.60	0.237	89.58	112.75
Stearic acid (g)	0.47	100.80	0.103	89.58	112.75
MUFA (g)	0.38	100.20	0.767	87.81	113.88
Oleic acid (g)	0.37	100.00	0.967	87.81	113.88
PUFA (g)	0.28	100.00	0.972	81.87	122.14
Linoleic acid (g)	0.29	100.00	0.999	81.06	123.37
Linolenic acid (g)	0.42	100.40	0.165	94.18	107.25
Arachidonic acid (g)	0.50	100.00	0.862	98.02	102.02
EPA (g)	0.59	100.00	0.868	98.02	102.02
DHA (g)	0.61	100.00	0.877	96.08	104.08
TFA (g)	0.44	100.00	0.927	92.31	108.33
Cholesterol (mg)	0.23	100.40	0.476	88.69	113.88
Proteins (g)	0.41	100.30	0.347	93.24	108.33
Arginine (g)	0.48	100.30	0.430	91.39	110.52
Cystine (g)	0.50	100.30	0.275	94.18	106.18
Phenylalanine (g)	0.48	100.50	0.194	92.31	109.42
Isoleucine (g)	0.47	100.50	0.162	92.31	109.42
Histidine (g)	0.49	100.30	0.360	92.31	109.42
Leucine (g)	0.40	99.70	0.738	82.70	119.72
Lysine (g)	0.40	99.60	0.607	82.70	119.72
Methionine (g)	0.52	100.20	0.622	91.39	109.42
Tyrosine (g)	0.49	100.50	0.209	92.31	109.42
Threonine (g)	0.49	100.40	0.254	92.31	109.42
Tryptophan (g)	0.52	100.20	0.541	93.24	107.25
Valine (g)	0.47	100.60	0.125	92.31	110.52
Carbohydrates (g)	0.43	100.80	0.001	96.08	106.18
Sugar (g)	0.51	101.11	0.022	90.48	112.75
Fructose (g)	0.51	101.71	0.053	82.70	125.86
Lactose (g)	0.60	100.90	0.298	81.87	124.61
Sucrose (g)	0.53	101.11	0.063	87.81	116.18
Starch (g)	0.31	101.01	0.094	87.81	116.18
Fiber (g)	0.46	100.80	0.068	91.39	111.63
Water (ml)	0.61	100.60	0.090	93.24	108.33
Calcium (mg)	0.45	100.40	0.338	91.39	110.52
Phosphorus (mg)	0.49	100.00	0.839	95.12	105.13
Iron (mg)	0.37	100.40	0.432	89.58	112.75
Magnesium (mg)	0.45	100.30	0.292	93.24	108.33
Vitamin A (RAE)	0.48	100.30	0.649	84.37	118.53
Thiamine (mg)	0.55	100.20	0.570	91.39	110.52
Riboflavin (mg)	0.45	100.40	0.322	92.31	109.42
Niacin (mg)	0.59	100.80	0.321	83.53	122.14
Vitamin B_6_ (mg)	0.46	100.30	0.446	90.48	110.52
Folate (μg)	0.52	100.40	0.453	89.58	112.75
Vitamin B_12_ (μg)	0.46	100.10	0.844	87.81	113.88
Vitamin C (mg)	0.57	100.80	0.140	88.69	113.88
Vitamin D (IU)	0.56	100.40	0.510	87.81	115.03
Vitamin E (mg)	0.51	100.20	0.694	91.39	109.42
Ethanol (g)	0.73	99.10	0.247	83.53	118.53
Caffeine (mg)	0.63	101.21	0.304	77.11	132.31

^a^Lower and upper Limits Of Agreement estimated through the Bland-Altman method.

SFA: saturated fatty acids; MUFA: monounsaturated fatty acids; PUFA: polyunsaturated fatty acids; EPA: eicosapentaenoic acid; DHA: docohexaenoic acid; TFA: trans fatty acids; RAE: retinol activity equivalents.

For almost all nutrients (48 out of 52) mean differences of intake estimates (FFQ1-FFQ2) were slightly positive, with an average mean ratio of 100.40%; the difference was significant only for carbohydrates and sugar (p < 0.05) (Table 5). LOA were narrow for all nutrients (Table 5), which showed good distribution of the differences in intake estimate around the mean intake.

**Figure 2:**
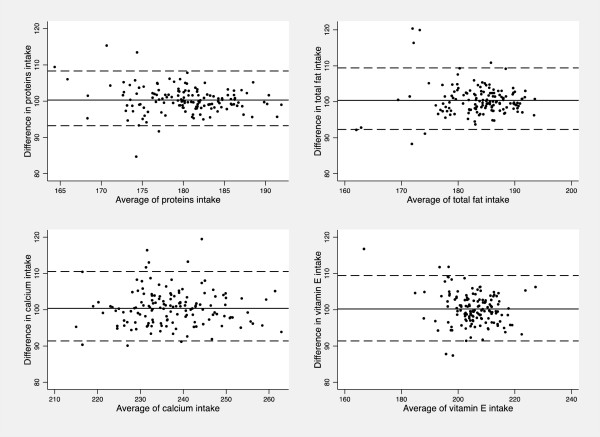
**Bland Altman plots for the reproducibility analysis of proteins, total fat, calcium and vitamin E.** The solid horizontal lines indicate the mean difference (percentage) between the two measures and the broken horizontal lines indicate the lower and upper Limits of Agreement (±t_91;0.025_SDs).

Arachidonic acid, cholesterol, cystine, carbohydrates, sucrose, starch, fiber, iron, magnesium, thiamine, riboflavin, vitamin B_6_ and vitamin B_12_ showed significantly higher differences at lower levels of average intake (p-value < 0.05); on the contrary, difference in DHA intake estimates was lower at lower levels of intake. Figure 2 shows scatter plots with LOA for proteins, total fat, calcium and vitamin E.

## Discussion

This study shows the relative and absolute reproducibility of the ASSO-FFQ in estimating 24 food groups, energy and 52 nutrients intake. Results from the analysis indicate that it is a reliable instrument for ranking individuals according to the level of intake.

The reproducibility was estimated by means of different tools. Based on the medians comparison, the intake estimates of all food groups, except for water, soft drinks, bread substitutes and savoury food, were not significantly different between the two FFQs, indicating high reliability of the estimation by the ASSO-FFQ. The results for foods such as milk and cheese, fruit, breakfast cereals, bread, fat spreads, fish/eggs/meat, pasta/rice, potatoes and vegetables are in line with the study from Matthys et al. [18]. The result for water is consistent with a previous study [18], which reported significant difference in the medians of water intake. The low reproducibility found for water in the present study is confirmed also by the kappa value and the percentage of subjects classified in the correct or adjacent quintile, which were among the lowest values obtained (0.32 and 63% respectively); moreover, the analysis on transformed data showed wide LOA, indicating low absolute reproducibility for water. This could be due to difficulties of adolescents in reporting water intake, since it is consumed many times a day and it is difficult to keep count of the right amount consumed. In American adolescents water intake is positively associated with age, and is inversely associated with the intake of beverage moisture and the energy density of foods [19]; maybe a further analysis of the association of water intake with different determinants in our sample could help better understanding water intake.

A similar argumentation could be done for soft drinks, whose consumption has significantly increased in the new generations, often leading them to substitute water intake.

The low reliability of bread substitutes and savoury food could be related to the specific inability of adolescents to count the daily intake of these food groups.The comparison of the mean differences of food intakes indicated also that estimates from the first administration were on average 0.15% higher than those from the second one, thus showing high agreement between the two FFQs. Moreover, all foods showed narrow LOA, indicating a good level of agreement between the two estimates, except for water, as mentioned above. Foods showed in Figure 1 were taken as an example, since results were similar for all foods; a comparison with other studies is not possible since the Bland Altman method has been rarely used in previous reproducibility studies for food intakes.

Also energy and all nutrients did not show significantly different medians, except for carbohydrates and sugar. The result for carbohydrates has been reported previously [20]. However, the relative reproducibility for carbohydrates and sugar was acceptable, as the classification in the same quintile was fair (respectively 30% and 28%), as well as the agreement estimated by the weighted kappa; moreover, ICC values showed fair to good reliability, and the LOA were narrow similarly to those of most nutrients. These results are comparable to those from a previous study [21], which found 36% (for carbohydrates) and 31% (for sugar) of subjects classified in the same quintile and reported high ICC for both nutrients (0.70 and 0.77 respectively). Another similar finding [10] showed 79% of subjects classified in one quintile and a substantial agreement (weighted kappa 0.64) for carbohydrates.

On average, subjects were quite well ranked according to the level of food intake (mean of correctly or adjacently classified 75%, and of correctly classified 40%).

Energy and nutrients showed also good relative reliability, with a percentage of correctly classified subjects ranging between 28% and 56% (mean 35%). These results are slightly higher than those reported previously by Dechamps et al. [21], ranging between 18% and 46% (mean 30%), and Watson et al. [22], ranging between 23% and 39% (mean 32%).

The analysis of weighted kappa showed moderate agreement both for food groups and energy/nutrients (mean kappa values were 0.47 and 0.48 respectively), thus indicating that the ASSO-FFQ has an overall acceptable reliability. Similar results were found for energy and nutrients in other studies [20, 21], which observed mean weighted kappa equal to 0.42 and 0.44. Good results were obtained also with the ICC values, which showed fair/good reliability for most food groups and most nutrients. In line with another study [20] total fat and iron were among the nutrients with poor reliability. On the contrary, a previous study [21] found higher reliability for total fat. However, for these two nutrients in the present study the difference of medians was not significant, the relative reproducibility assessed by the quintiles method was acceptable, the agreement was fair and the LOA were narrow (Figure 2 includes LOA for total fats), thus indicating a reasonable reproducibility.

Bland Altman analysis showed very small mean differences and narrow LOA both for food groups and nutrients, indicating an absolute reliability between the two measures. Moreover, a trend towards smaller difference in some food groups and nutrients according to increased intake values was assessed, so that the level of absolute reliability of the ASSO-FFQ was related to the average level of intake estimates.

Common outcome of many studies [20, 23–25] is that the reported intakes are generally higher in the first administration than in the second. The present study showed that intake estimates from the first administration were on average 0.4% higher than those from the second one. These findings are in line with those studies, even though significant differences were found only for few foods and nutrients.

All the obtained results lead to state that the ASSO-FFQ is a reliable tool. Although gathered measures could be biased by the self-reporting method of the ASSO-FFQ, there is evidence that children are more accurate reporters than their parents [26]. Moreover, as suggested previously [27], the second ASSO-FFQ was administered after one month, an interval that was retained reasonable to avoid change in diet due to food seasonality; other studies considered a longer time interval [8, 22].

However, the study suffers from some limitations. Firstly, the sample was composed of a higher number of male adolescents compared to females; this was due to the predominant presence of males in one of the selected schools. Another limitation was the assumption that for test–retest reliability the true intake did not change between administrations [6]. The other assumption was that the time period between administrations was not too long, in order to avoid any changes in diet or recall bias, and not too short, in order to avoid that subjects could reproduce the answers by mean of learning processes [28].

## Conclusions

In conclusion, this reproducibility study provides information on the consistency and stability of the answers of a previously validated FFQ. The ASSO-FFQ is a reliable instrument for estimating food groups, energy and nutrients intake in adolescents, and thus can be used in epidemiological studies on large scale to obtain reliable estimations over time.

## Authors’ information

João Breda is staff members of the WHO Regional Office for Europe. The author alone is responsible for the views expressed in this publication and they do not necessarily represent the decisions or the stated policy of the World Health Organization.

## Electronic supplementary material

Additional file 1: **Food groups and included food items, used for the reproducibility assessment.** (DOCX 146 KB)

## Abbreviations

FFQ: Food frequency questionnaire
ASSO: Adolescents and surveillance system for the obesity prevention
WFR: Weighted food record
SFA: Saturated fatty acids
MUFA: Monounsaturated fatty acids
PUFA: Polyunsaturated fatty acids
EPA: Eicosapentaenoic acid
DHA: Docohexaenoic acid
TFA: Trans fatty acids
RAE: Retinol activity equivalents
ICC: Intraclass correlation coefficient
LOA: Limits of agreement.
