# Supplementary material for: The web-based ASSO-food frequency questionnaire for adolescents: relative and absolute reproducibility assessment
Source: Nutr J. 2014 Dec 17;13:119. doi: 10.1186/1475-2891-13-119 (PMC4320587; doi:10.1186/1475-2891-13-119)
Supplement: Supplementary file 1 — Additional file 1: Food groups and included food items, used for the reproducibility assessment. (DOCX 146 KB) [file 12937_2014_867_MOESM1_ESM.docx]

**Additional file. Food groups and included food items, used for the reproducibility assessment.**

| **Food groups** | **Food items** |
| --- | --- |
| Vegetables | Mixed salad (carrots, fennels, lettuce, tomatoes, radish, etc.), cooked leaf vegetables (chicory, endive, lettuce, radish, rocket, spinach, etc.), cooked non-leaf vegetables (artichokes, aubergines, broccoli, carrots, cauliflower, cucumber, fennels, mushrooms, peppers, pumpkins, zucchini, etc.), soup or potage (without pasta or rice). |
| Fresh fruit | Fresh fruit (apricots, apples, bananas, figs, grape, grapefruit, kiwi, mandarins, melons, oranges, peaches, pears, plums, watermelon, etc.), fresh fruit as soft fruit (blackberries, blueberries, cherries, strawberries, raspberries, etc.). |
| Dried fruit | Apricots, dates, figs, grape, plums, etc. |
| Nuts | Almonds, hazelnuts, peanuts, pistachios, walnuts, etc. |
| Legumes | Beans, broad beans, chickpeas, green beans, lentils, peas, split soy, etc. |
| Breakfast cereals | Corn flakes, muesli, puffed rice, etc. |
| White bread | White bread. |
| Bread substitutes | Wheat or brown bread, sandwich loaf, crackers, rusks, breadsticks, puffed rice rusks. |
| Pasta/rice/couscous | Pasta, rice, couscous |
| Potatoes | Packed fries, fries, boiled or oven-baked potatoes, mashed potatoes, potato dumplings. |
| Sweets | Milk or dark chocolate, chocolate cream (such as Nutella), Nesquik, cocoa powder, jam/marmalade, honey, sugar, brown sugar, fruit sugar, sweetener (acesulfame k, saccharin, aspartame, sucralose, cyclamate, etc.), shortbreads, cookies, cakes, tarts, croissants, brioches, muffins, sweet with cream/custard (cakes with cream/custard/chocolate cream, pudding etc.), sweet crepes, packed snacks, bars (kit-kat, mars, sneakers, etc.), fresh ice-creams, packed ice-creams, candies and chewing gum. |
| Cheeses/yogurt | Yogurt, soft cheeses (ricotta cheese, soft cheese, cream cheese, etc.), medium-matured cheese (mozzarella, scamorza cheese, Gorgonzola cheese, Brie, emmenthal cheese, Gruyere, etc.), matured cheese (ripened cheese, sheep milk cheese, Parmesan, etc.), grated cheese. |
| Fishery products | Blue fish (sardines, herrings, mackerels, anchovies, etc.), fish (grouper, sea bream, cod, sole, black sea bass, lake bass, fresh salmon, white bream, tuna, etc.), shellfishes (squids, mussels, clams, octopus, shrimps, lobsters, scampi, etc.), canned fish (tuna, salmon, mackerel, sardines, etc.). |
| Meat | Red meat (beef, sheep, goat, equine, buffalo, goose, venison, pheasant, etc.), white meat (veal, mutton, pork, lamb, poultry, turkey, rabbit, etc.), pork and sausage meat, offal (spleen, liver, bowel, tripe, kidney, brain, etc.), cold cuts (steak o raw ham, speck ham, dried beef, salami, mortadella, bacon, wurstel, turkey breast, etc.), canned meat. |
| Eggs | Eggs. |
| Animal fats | Butter cream, béchamel, mayonnaise. |
| Oils | Extra virgin olive oil/olive oil, oilseed (peanut, sunflower, corn, etc.), margarine. |
| Savoury food | Pizza, ravioli (ravioli, tortellini, etc.), popcorn, fast food meal (cheeseburger, hamburger, hot-dog, etc.), piadina, savoury crepe, convenience food (ready-to-eat dishes, pre-cooked foods, frozen food, etc.), savoury pies, quiche. |
| Water | Still water, sparkling water. |
| Soft drinks | Soft drinks (Coca-Cola, Pepsi, Fanta, Sprite and similar), light soft drinks (coca-zero, coca-light and similar), energy drinks (red bull, burn, atomic, dynamite no fear, extreme, etc.). |
| Fruit juice | Canned fruit or vegetable juices (canned vegetable juices: carrot, tomato juices, etc.), freshly squeezed fruit or vegetable juices. |
| Milk | Whole milk, semi-skimmed milk, skimmed milk, milk not from cow (she-ass, buffalo, goat, sheep), vegetable milk (soy, rice, almond, oat, coconut). |
| Tea/coffee | Sugared tea, non-sugared tea, sugared coffee, non-sugared coffee. |
| Alcoholic drinks | Slightly alcoholic soft drinks (drinks such as Bacardi breeze, Eris toff ice, Campari Mix, etc.), beer, red wine, white wine, liqueurs (lemon liqueur, elderberry, bitter, cream (lemon or mandarin liqueur, and similar, etc.), etc.), spirits (grappa, cognac, vodka, whiskey, rum, gin, brandy, etc.), cocktails (mojito, metropolitan, Negroni, rum and coca, etc.). |
